# Relationships of apelin concentration and *APLN* T-1860C polymorphism with obesity in Thai children

**DOI:** 10.1186/s12887-020-02350-z

**Published:** 2020-09-30

**Authors:** Kanjana Suriyaprom, Banchamaphon Pheungruang, Rungsunn Tungtrongchitr, Orn-uma Y. Sroijit

**Affiliations:** 1 Faculty of Medical Technology, Rangsit University, Paholyothin Road, Mueang Pathum Thani district Pathum Thani, 12000 Thailand; 2 Department of Tropical Nutrition & Food Science, Faculty of Tropical Medicine, Mahidol University, 420/6 Rajvithi Road, Rajthevee, Bangkok, 10400 Thailand

**Keywords:** Apelin, Gene polymorphisms, Cardiometabolic parameters, Obese children, Thai

## Abstract

**Background:**

Childhood obesity represents a serious global health crisis. Apelin and its receptor system are widely distributed throughout the central nervous system and have been demonstrated to serve a role modulating feeding behaviour and energy homeostasis. The purposes of this study were to examine apelin concentrations and anthropometric-cardiometabolic parameters in obese and non-obese children and to identify associations of *APLN* T-1860C and *APLNR* G212A polymorphisms with apelin levels and obesity among Thai children.

**Methods:**

This case-control study included an analysis of 325 Thai children: 198 children with obesity and 127 healthy non-obese children. Anthropometric-cardiometabolic variables and apelin concentration were measured. Genotyping of *APLN* T-1860C and *APLNR* G212A was performed using the polymerase chain reaction-restriction fragment length polymorphism technique.

**Results:**

The obese group had significantly lower apelin and HDL-C levels but significantly higher triglycerides and glucose (TyG) index values, TG/HDL-C ratio and TC/HDL-C ratio than the non-obese group (*p* < 0.01). Apelin level was negatively correlated with body size phenotypes and cardiometabolic parameters (*p* < 0.05). The *APLN* T-1860C polymorphism (OR = 4.39, 95% CI = 1.25–15.28) and apelin concentration (OR = 0.45, 95% CI = 0.23–0.92) were significantly associated with obesity among female children (*p* < 0.05) only, after adjusting for potential covariates. However, the *APLNR* G212A polymorphism showed no significant relationship with apelin concentration or obesity.

**Conclusion:**

These findings in Thai children suggest that apelin concentrations are related to obesity and cardiometabolic parameters. Furthermore, the *APLN* T-1860C polymorphism may influence susceptibility to obesity among female children.

## Background

Childhood obesity is a serious health threat to children since obesity in the early stages of life raises the risk of obesity in later life, with the enhanced risk for developing non-communicable diseases. Thailand now has one of the highest levels of obesity prevalence in Asia, with overweight and obesity among school-aged children being 8.98 and 7.26%, respectively [1]. Obesity is considered a multifactorial disease due to the interaction of genes, environment and lifestyle [2]. However, the mechanism of obesity development is not yet fully understood.

Apelin, a member of adipose tissue-derived peptides, encodes a novel peptide that acts through its G protein-coupled apelin receptor [3]. Apelin and its receptor are widely distributed throughout the central nervous system and peripheral tissues that are proficient in controlling feeding behaviour, energy homeostasis and neuroendocrine functions [4]. Existing evidence—especially in children—concerning apelin and its relation to obesity and cardiometabolic risk factors remain less clear, with published results being inconsistent. Some studies have reported increased apelin levels in obese children when compared to a normal weight group [5], while other studies found no significant relationships [6] or even lower apelin levels in obese compared to non-obese children [7, 8]. However, we hypothesised that apelin concentrations may be associated with obesity and cardiometabolic risk factors in Thai children.

The apelin gene (*APLN*) is localised on the X chromosome at Xq25-q26.1 [3], a region close to a susceptibility locus for obesity that was identified by linkage mapping in the African Americans [9], while the apelin receptor gene (*APLNR*) is located on chromosome 11q12 [4]. The *APLN* T-1860C polymorphism (rs56204867), located in the promoter region, was first identified in the Han Chinese population and linked with the susceptibility to cardiovascular risk factors, including hypertension [10]. However, only one previous study has provided data on the relationship between the *APLN* T-1860C polymorphism and obesity in Chinese women [11]; however, it lacked proper information on this polymorphism in children. The *APLNR* G212A (rs11544374) gene occurs in the 5′ untranslated region and this polymorphism is linked to circulating apelin levels [7] and cardiovascular risk [12, 13]. However, previous studies have produced conflicting results and few reports have assessed the relationship between these gene polymorphisms and obesity in children [7]. Therefore, the present study aimed to examine concentrations of apelin and anthropometric-cardiometabolic parameters in obese and non-obese children and to search for associations of *APLN* T-1860C and *APLNR* G212A polymorphisms with apelin levels and obesity among Thai children. To the best of our knowledge, this is the first study to evaluate these associations in Thai children.

## Methods

Following approval by the Ethical Committee of Research Institute of Rangsit University (RSEC 28/2560), Thailand and in accordance with the Declaration of Helsinki, the purposes of the study were carefully explained by both the parents and voluntary children before obtaining written informed consent from parents or children. The present observational case-control study consisted of 325 Thai children, aged 8–13 years living in urban and suburban residential areas of Bangkok, Thailand. Among them, 198 obese children (107 male, 91 female) and 127 healthy non-obese children (62 male, 65 female) were chosen during a health screening programme among check-up subjects in November 2018–July 2019. The sample size of our study was calculated based on a study by Ba et al. [6] with regarding the standard deviation (SD) of apelin levels (0.43 ng/ ml). To detect a true potential difference of 0.35 ng/ml in the apelin levels between the compared groups (statistical power: 80%, type I error = 0.05). Physical examinations included an assessment of the presence of acanthosis nigricans on and around the neck and a medical history check for each subject, which were conducted by the same medical doctor throughout the study. Subjects with a history of liver, kidney, cardiovascular, infectious or inflammatory diseases and those that use prescription medication for any reason were excluded from this study.

### Anthropometric measurements

The body weight of each subject was measured to the nearest 0.1 kg using a carefully calibrated beam balance (Detecto®, Cardinal Detecto Scale Manufacturing, USA). Height was measured to the nearest 0.5 cm using a vertical measuring rod. Children were instructed to stand on the balance and look straight with light clothing and without footwear. Body mass index (BMI) was defined as weight (kg)/height (m^2^) and transformed into BMI z-scores that were adjusted for gender and age according to the new World Health Organization reference [14]. Cut-off values for weight status were as follows: obese BMI z-score > 2SD and normal weight BMI z-score ≤ 1SD to > − 1SD. Waist circumference (WC) was also measured to the nearest 0.5 cm at the midpoint between the top of the iliac crest and the lower margin of the last palpable rib using a flexible measuring tape. Blood pressure (BP) was measured three times by a nurse after 5 to 10 min’ rest in the seated position.

### Laboratory measurements

Eight millilitres of venous blood was taken from subjects in the morning between 7:00 AM and 9:00 AM after a 12-h overnight fast. Glucose and lipid profile (triglycerides [TG], total cholesterol [TC], low-density lipoprotein cholesterol [LDL-C] and high-density lipoprotein cholesterol [HDL-C]) were measured using enzymatic methods on Roche Integra Biochemical analyser with commercially available kits (Roche Diagnostics GmbH, Mannheim, Germany). The TG and glucose index (TyG) was calculated as: ln[fasting TG (mg/dl) × fasting plasma glucose (mg/dl)/2], according to previous studies and the index is expressed by a logarithmic scale [15]. Plasma apelin level was determined using the enzyme-linked immunosorbent assay (ELISA) protocol according to the manufacturer’s instructions (Human apelin-12 ELISA kit, Phoenix Pharm, Burlingham, CA, USA). The minimal detectable concentrations of apelin were 0.031 μg/ml. The intra- and inter-assay errors were approximately < 5 and < 10%, respectively.

### Polymerase chain reaction-restriction fragment length polymorphism (PCR-RFLP) technique

DNA was extracted from EDTA-treated whole blood using a FlexiGene DNA kit (Qiagen, Hilden, Germany). The genotyping of *APLN* T-1860C and *APLNR* G212A was performed using PCR-RFLP assay. DNA fragments were amplified by PCR (PE Applied Biosystems, Foster City, CA, USA). For analysis of the *APLN* T-1860C polymorphism, the following primers were used:

Forward primer – 5′-GGGGAACAGTGAAGGGAGAATGGT-3′.

Reverse primer – 5′-AGAAGCGGGTCCTGAAGTTGTTTG-3′.

A 50 μl PCR reaction was performed according to the protocol described by Niu et al. [16]. For analysis of the *APLNR* G212A polymorphism, the following primers were used:

Forward primer – 5′-TTCTGCAGGAGACAGGCTTC-3′.

Reverse primer – 5′-GGCAGACAACCAGTCTGAGT-3′.

A 50 μl PCR reaction was conducted according to the protocol adapted from Kotanidou et al. [7]. PCR products were detected on 2% agarose gel containing ethidium bromide. Aliquots of the PCR products were digested with 10 U *XhoI* and *DdeI* restriction enzymes for the APLN T-1860C and *APLNR* G212A polymorphisms, respectively. The *APLN* T-1860C polymorphism was identified by the presence of a single 732 bp band indicating the TT genotype, a single 621 bp band indicating the CC genotype, and two zones (732 bp, 621 bp) indicating the CT heterozygous genotype. Furthermore, the *APLNR* G212A polymorphism was identified by the presence of a single 157 bp band indicating the GG genotype, a single 203 bp band indicating the AA genotype, and two zones (157 bp, 203 bp) indicating the GA heterozygous genotype. The sizes of restriction fragments were visualised by electrophoresis through an ethidium bromide-stained 2% agarose gel.

### Statistical analysis

Statistical analysis was performed using SPSS for Windows version 11.5 (SPSS, Chicago, IL). The normality of the sample distribution of each continuous variable was tested using the Kolmogorov-Smirnov test. Continuous variables were expressed as mean ± standard deviation (SD) or median with interquartile range (25th–75th percentile) in the presence of an abnormal distribution. For comparisons between the obese and non-obese groups, the independent samples t-test was used for parameters with normal distribution, while the Mann-Whitney U-test was used for the parameters with non-normal distribution. Allele and genotype frequencies between the two groups were compared using the chi-squared (χ2) test. The Minitab statistical computer program was used to calculate the odds ratio (OR) and 95% confidence interval (CI). Correlation between variables was assessed by Spearman’s rho. To assess links between obesity as a dependent variable and other potential factors, binary logistic regression was applied. The level of statistical significance was set at *P* < 0.05. The goodness-of-fit of binary logistic regression models was established by the Hosmer-Lemeshow test.

## Results

Total of 325 children were enrolled in this study, including 198 obese children (91 girls [46.0%]/107 boys [54.0%]) and 127 non-obese children (65 girls [51.2%]/62 boys [48.8%]). Table 1 summarises the demographic and clinical characteristics between non-obese and obese children. The age difference between the non-obese group (10.0 ± 1.5 years) and the obese group (10.3 ± 1.30 years) was not significant (*P* > 0.05). Acanthosis nigricans was observed in approximately 30.8% of the obese group and was not observed in the non-obese group (*P* < 0.01). Plasma apelin levels were significantly lower in the obese group than the non-obese group (*P* < 0.01), while higher body size phenotypes including weight, BMI, BMI z-score and WC were observed in the obese group when compared to the non-obese group (*P* < 0.01). Moreover, significant differences were observed between the obese and non-obese groups in terms of cardiometabolic parameters, including TyG index, HDL-C, TG, and the ratios of TC/HDL-C, TG/HDL-C and LDL-C/HDL-C (*P* < 0.01). When males and females were analysed separately (see Supplementary file; Table S1), obese girls had significantly lower apelin than non-obese girls, and this result was consistent in boys. Spearman’s rank correlation test results are presented in Table 2. Apelin level was negatively correlated with weight, BMI, BMI z-score, WC, BP, TyG index, TG, and the ratios of LDL-C/HDL-C, TC/HDL-C and TG/HDL-C (*P* < 0.05) (see Table 2).

**Table 1:** Comparison of the demographic and clinical characteristic between non-obese and obese children

Variables	Non-obese group (*n* = 127)	Obese group (*n* = 198)	*P*-value
Age (years)	10.0 ± 1.5	10.3 ± 1.3	0.109
Weight (kg)	35.8 ± 9.3	62.4 ± 15.6	< 0.001**
BMI (kg/m^2^)	17.3 (15.9, 18.9)	27.2 (24.6, 29.7)	< 0.001**
BMI z-score	0.3 (−0.3, 0.7)	2.8 (2.4, 3.3)	< 0.001**
WC (cm)	62.1 ± 7.2	86.5 ± 12.7	< 0.001**
Systolic BP (mmHg)	100.0 ± 11.0	115.0 ± 12.0	0.065
Diastolic BP (mmHg)	65.1 ± 9.0	70.0 ± 9.0	0.109
Apelin (ng/ml)	1.6 (0.9, 2.4)	1.0 (0.6, 1.6)	< 0.001**
Glucose (mg/dl)	88.0 ± 5.6	90.0 ± 6.0	0.506
TyG index	7.61 ± 0.30	8.28 ± 0.44	0.002**
TC (mg/dl)	173.0 ± 30.0	176.0 ± 31.0	0.438
TG (mg/dl)	58.0 (42.0, 67.0)	85.0 (66.0, 118.0)	< 0.001**
HDL-C (mg/dl)	55.0 (46.0,64.0)	44.0 (38.0–50.0)	< 0.001**
LDL-C (mg/dl)	103.0 ± 23.0	111.0 ± 26.0	0.270
TG/HDL-C	0.9 (0.7, 1.3)	2.0 (1.4, 2.9)	< 0.001**
TC/HDL-C	3.01 ± 0.49	4.07 ± 0.94	< 0.001**
LDL-C/HDL-C	1.89 ± 0.39	2.60 ± 0.78	< 0.001**
Acanthosis nigricans			
Yes	0.0 (0%)	61 (30.8%)	< 0.001 ^a^**
No	127 (100%)	137 (69.2%)	

Data are means ± standard deviation or medians with interquartile range (25th–75th percentile) Significance levels: **P < 0.01

^**a**^Based on the results of a chi-squared test

**Table 2:** Correlation coefficients of apelin concentration with other parameters, in all subjects

Variables	Apelin
Age	−0.099
Weight	−0.291**
BMI	−0.298**
BMI z-score	−0.281**
WC	−0.320**
Systolic BP	−0.241**
Diastolic BP	−0.235**
Glucose	−0.054
TyG index	−0.171*
TC	−0.072
TG	−0.160*
HDL-C	0.109
LDL-C	−0.091
TC/HDL-C	−0.150*
LDL-C/HDL-C	−0.143*
TG/HDL-C	−0.171*

Significance levels: * *P* < 0.05, ** *P* < 0.01

To evaluate the associations of single-nucleotide polymorphisms (SNPs), *APLN* T-1860C and *APLNR* G212A with apelin level and anthropometric-cardiometabolic parameters, we performed a multivariate analysis. When *APLN* T-1860C and *APLNR* G212A were used as a dependent variable, the results showed no significant associations of these two polymorphisms with apelin concentration and anthropometric-cardiometabolic parameters (*P* > 0.05) after adjusting for potential covariates including age, sex and acanthosis nigricans (see Supplementary file; Table S2). The genotypic and allelic frequency results of *APLN* T-1860C and *APLNR* G212A polymorphisms in the obese and non-obese groups are presented in Table 3. All participants were genotyped for the SNPs *APLN* T-1860C and *APLNR* G212A; genotype frequencies were not significantly different between the non-obese and obese groups for these two gene polymorphisms, with *P*-values of 0.325and 0.121, respectively. The minor allele frequencies (MAF) of the whole Thai population for the SNPs *APLN* T-1860C and *APLNR* G212A were 33.8 and 13.5%, respectively. However, this study was also analysed by gender due to the *APLN* gene located on the X chromosome. Among female children, significant differences in the genotypic and allelic frequencies of the *APLN* T-1860C polymorphism were observed between the non-obese and obese groups (*P* < 0.05). This polymorphism showed a significant link to obesity (OR = 2.05, *P* < 0.05) and the CC genotype frequencies in the obese and non-obese groups were 18.7 and 7.7%, respectively. In contrast, the *APLN* T-1860C polymorphism showed no significant link to obesity in male children (*P* > 0.05). Regarding links between *APLNR* G212A polymorphism and obesity phenotype, the genotypic and allelic distributions of this gene polymorphism between the obese and normal weight groups were not significantly different, and the *APLNR* polymorphism at G212A was not related to obesity in both males and females (*P* > 0.05). Moreover, there were no significant deviations from Hardy–Weinberg equilibrium (HWE) for the SNPs *APLN* T-1860C and *APLNR* G212A in non-obese females (*P* = 0.856 and 0.108, respectively), obese females (*P* = 0.503 and 0.594, respectively), non-obese males (*P* = 0.274 for *APLNR* G212A) and obese males (*P* = 0.612 for *APLNR* G212A).

**Table 3:** Genotypic and allelic distribution of *APLN* T-1860C and *APLNR* G212A polymorphisms in non-obese and obese children

*APLN* T-1860C	Obese *n* (%)	Non-obese *n* (%)	Genotypic or allelic *P*-value
Total: genotype			
CC	52 (26.3)	25 (19.7)	
CT	41 (20.7)	25 (19.7)	0.325
TT	105 (53.0)	77 (60.6)	
TT vs CT + CC^**a**^	1.36 (0.87–2.15)		
	[0.170]		
C allele	145 (0.366)	75 (0.295)	0.062
T allele	251 (0.634)	179 (0.705)	
Female: genotype			
CC	17 (18.7)	5 (7.7)	
CT	41 (45.0)	25 (38.5)	0.042*
TT	33 (36.3)	35 (53.8)	
TT vs CT + CC^**a**^	2.05 (1.08–3.92) [0.029]*	Reference	
C allele	75 (0.412)	35 (0.269)	0.009**
T allele	107 (0.588)	95 (0.731)	
Male: genotype			
CC	**-**^**b**^	**-**^**b**^	
CT	**-** ^**b**^	**-** ^**b**^	
TT	**-** ^**b**^	**-** ^**b**^	
T vs C^**a**^	1.02 (0.52–2.00) [0.902]	Reference	
C allele	70 (0.327)	40 (0.323)	0.931
T allele	144 (0.673)	84 (0.677)	
*APLNR* G212A	Obese *n* (%)	Non-obese *n* (%)	Genotypic or allelic *P*-value
Total: genotype			
AA	3 (1.5)	4 (3.2)	0.121
AG	52 (26.3)	22 (17.3)	
GG	143 (72.2)	101 (79.5)	
GG vs AA+AG^**a**^	1.49 (0.88–2.54)	Reference	
	[0.137]		
A allele	58 (0.146)	30 (0.118)	0.303
G allele	338 (0.854)	224 (0.882)	
Female: genotype			
AA	1 (1.1)	2 (3.1)	0.297
AG	22 (24.2)	10 (15.4)	
GG	68 (74.7)	53 (81.5)	
GG vs AA+AG^**a**^	1.49 (0.68–3.28) [0.310]	Reference	
A allele	24 (0.132)	14 (0.108)	0.432
G allele	158 (0.868)	116 (0.892)	
Male: genotype			
AA	2 (1.9)	2 (3.2)	
AG	30 (28.0)	12 (19.4)	0.410
GG	75 (70.1)	48 (77.4)	
GG vs AA+AG^**a**^	1.43 (0.70–2.96) [0.330]	Reference	
A allele	34 (0.159)	16 (0.129)	0.456
G allele	180 (0.841)	108 (0.871)	

^**a**^Values are the odds ratio with 95% confidence interval in parentheses; *P −* values in brackets

^**b**^Since the apelin gene is on the X chromosome (only one copy), it is irrelevant to present the genotype data for males

Significance levels: * *P* < 0.05; ** *P* < 0.01 based on the results of a chi-squared test

Logistic regression results for possible associations of obesity and circulating apelin concentration, SNPs *APLN* T-1860C and *APLNR* G212A after controlling for potential factors are presented in Table 4. Among females, the *APLN* T-1860C polymorphism (OR = 4.39, 95% CI = 1.25–15.28) and apelin concentration (OR = 0.45, 95% CI = 0.23–0.92) were significantly associated with obesity (*P* < 0.05). However, among males, there were no significant associations of these variables with obesity (*P* > 0.05). The Hosmer-Lemeshow goodness-of-fit test in females (χ^2^ = 0.777, *P* > 0.05) and in males (χ^2^ = 0.990, *P* > 0.05) was not statistically significant, while the fit between the predictive model and the data was acceptable**.**

**Table 4:** Logistic regression analysis when obesity was used as a dependent variable and apelin concentration, *APLN* T-1860C, and *APLNR* G212A polymorphisms were taken as independent variables

Variables	β	Adjusted OR^a^	95% CI	*P*-value
Female:				
*APLN* T-1860C	1.48	4.39	1.25–15.28	0.020*
*APLNR* G212A	0.43	1.53	0.37–6.60	0.566
Apelin	−0.78	0.45	0.23–0.92	0.029*
Male:				
*APLN* T-1860C	−0.41	0.66	0.27–1.65	0.377
*APLNR* G212A	0.26	1.30	0.45–3.71	0.626
Apelin	−0.27	0.75	0.45–1.24	0.269

^**a**^Value is the odds ratio adjusted for sex, age and acanthosis nigricans

95% CI is the 95% confidence interval of the odds ratio

Significance level: * *P* < 0.05

## Discussion

To the best of our knowledge in the Thai population, the present study supports our hypothesis that circulating apelin levels are lower in obese Thai children, while the presence of the *APLN* T-1860C polymorphism increases the risk of obesity among female children. Obesity is a multifactorial disease and its development is attributed to the misregulation of energy balance, genetic predisposition, and social and environmental factors [2]. Understanding childhood obesity is important for reducing its incidence and the development of related cardiometabolic disorders. Our findings are in agreement with previous studies on obese children and adolescents in Greece [7], as well as pubertal obese children in Turkey [8], in that obese children had significantly decreased plasma apelin concentrations compared to children with normal weight. In the same way, Reinehr et al. found a trend in Germany toward lower apelin levels in obese children when compared to lean children (*P* = 0.06) [17]. However, in contrast to our findings, a previous study in Egyptian children showed that apelin was significantly higher in obese versus non-obese children [5], which is in agreement with the findings of Ziora et al. on Polish girls with obesity [18]. However, the findings of Soriguer et al. suggest that elevated plasma apelin concentrations were only evident in obese people with glucose intolerance or diabetes but not among obese people with normal glucose [19]. On the other hand, no significant differences in apelin levels were observed between obese and non-obese Chinese boys [6]. Table 5 shows the comparison of demographic data and circulating apelin changes between obese children and non-obese children in various studies. Notably, these inconsistent findings related to apelin concentrations among existing studies may be influenced by differences among populations in terms of ethnicity, the severity of obesity, sample size and study design. Furthermore, differences in sources of specimen (serum/plasma) or test kits for the measurement in all apelin forms or only in isoforms may explain inconsistent results about apelin levels. In the present study, apelin-12 EIA kit presents 100% cross-reactivity with human apelin-12, apelin-13 and apelin-36. Concerning sample size, the sample size of children in the present study is larger than those of existing studies (see Table 5). However, children may have not yet developed obesity-related metabolic diseases. While apelin is involved in the regulation of food intake, energy metabolism, cardiovascular system and neuroendocrine functions, it has also been described as a beneficial adipokine related to obesity functions [4, 20]. Furthermore, study of Henley et al. revealed the change of apelin secretory dynamics in response to oral glucose and lack of an apparent circadian variability [21]. Apelin is related to glucose and lipid metabolism by the phosphorylation of the AMP-activated protein kinase (AMPK) and enhancing insulin sensitivity [20, 22]. Several mechanisms have been proposed to investigate the role of apelin in resisting obesity. Firstly, apelin may regulate appetite and decrease food intake behaviour through its abundant receptors in the hypothalamus [4, 23]. Secondly, the effect of apelin on energy metabolism may be an important factor of the apelin-induced reduction in adiposity by increased uncoupling protein (UCP)-1 mRNA and protein expression in brown adipose tissue, leading to increased energy expenditure [20]. Moreover, Sawane et al. demonstrated that apelin signalling promotes lymphatic and blood vessel integrity and blocks the increased permeability of endothelial cells induced by dietary fatty acids, resulting in the inhibition of fat accumulation and attenuation of obesity [24]. Therefore, the findings from the present study are in line with these previous studies that low levels of apelin are likely to lead to obesity. Moreover, our findings are consistent with previous studies demonstrating that there was an inverse correlation between apelin concentration and body size phenotypes [7, 25], whereas one other study failed to identify such relationships [17]. Yue et al. showed that mice deficient in apelin signalling had increased circulating levels of free fatty acids and glycerol along with increased adiposity since high levels of free fatty acid could lead to insulin resistance, which may help to explain the beneficial role of apelin in regulating metabolic homeostasis [22]. Furthermore, it has been reported that apelin is associated with multiple physiological processes in the cardiovascular system; thus, it may act via central nervous system mechanisms to regulate peripheral vascular function [26]. In vivo and in vitro rodent models have also shown the involvement of apelin in direct cardioprotective actions involving the reperfusion injury salvage kinase (RISK) pathway and mitochondrial permeability transition pore (MPTP) regulation [27]. Our data from Thai children support the existence of negative relationships between apelin concentration and parameters that indicate cardiometabolic risk, including BP, TyG index, and ratios of TG/HDL-C, TC/HDL-C and LDL-C/HDL-C. As a marker of identifying insulin resistance with high sensitivity and specificity, TyG index had also been reported as being associated with cardiovascular disease risk [15]. Additionally, TC and TG are predictors of the balance of lipid metabolism, while a previous study also noted that individuals with high TC/HDL-C or LDL-C/HDL-C ratios had greater cardiovascular risk [28] because these ratios reflected the balance between transport of cholesterol to peripheral tissues and reverse transport to the liver. Furthermore, a recent study in a rat model showed that apelin improved dyslipidaemia by decreasing TC, TG and LDL-C, and increasing HDL-C concentration [29]. Overall, our findings in Thai children suggest that apelin might provide an inverse association with cardiometabolic risks. Moreover, a decrease in circulating apelin level in obese children suggest that apelin homeostasis is impaired in the obese state, which could be a warning sign before the emergence of well-known obesity-related metabolic diseases and requires further long-term studies. However, since exercise training upregulates muscle apelin expression in obese subjects, increasing the levels of physical activity among obese children could be an alternative and beneficial pathway to prevent obesity-related metabolic diseases [30].

**Table 5:** Comparison of demographic data and circulating apelin changes between obese children and non-obese children

	Ethnicity	Sample size (obese/ non-obese)	Age	Sources and types of apelin measurement	Levels of apelin
The present study	Thai children	198/127	10.5/10.1	Apelin-12 in plasma	↓^*^
Kotanidou et al. [7],	Greek children and adolescents	90/90	11.6/11.7	Apelin-12 in serum	↓^*^
	Greek children	45/45	9.3/8.4		↓^*^
	Greek adolescents	45/45	13.1/15.2		↓^a^
Reinehr et al. [17],	German children	80/40	10.9/11.6	Apelin in serum	↓^b^
Tapan et al. [8],	Turkish children	32/40	11.0/12.0	Apelin-12 in serum	↓^*^
	Pubertal children	16/21	13.2/13.2		↓^*^
	Pre-pubertal children	16/19	8.8/8.7		↔
El Wakeel et al. [5],	Egyptian children	50/31	9.5/8.7	Apelin in serum	↑^*^
Ziora et al. [18],	Polish girls	30/61	14.6/15.4	Apelin-12 & Apelin-36 in serum	↑^*^
Ba et al. [6],	Chinese girls	20/16	9.7/10.9	Apelin-12 in serum	↑^*^
	Chinese boys	28/24	10.9/10.9		↔

^Significance level: *^ *P* < 0.050

^a^*P* = 0.051, ^b^ *P* = 0.061

Both apelin and its receptor have been implicated as key mediators of physiological responses to multiple homeostatic processes. Although molecular studies on *APLN* and *APLNR* genes have been performed, the relationships of these genes with obesity remain limited, especially in children. The *APLN* gene is located on chromosome Xq25–26.1 [3]. Furthermore, the *APLN* T-1860C polymorphism (rs56204867), located in the promoter region, was first linked to hypertension susceptibility [10]. The rs56204867 was analysed in our study and the minor allele frequency (MAF) for this SNP in our study population (33.8%) paralleled to the previous studies which represented the MAF from Han Chinese (34.7%) [10], East Asian (37.2%), African (31.1%) and Korean population (31.1%) [31]. To our knowledge, the first study of the relationship between *APLN* T-1860C polymorphism and apelin level was conducted by Akcılar et al. in the Turkish population [13]. They observed no significant difference between the plasma apelin levels of healthy controls and coronary artery disease patients with different genotypes (*P* = 0.223 and *P* = 0.667, respectively), while individuals with the CC genotype had an approximately six times greater risk of coronary artery disease when compared to those carrying the TT genotype. Similarly, our results confirm that the *APLN* T -1860C polymorphism is not linked with apelin levels, whereas the C allele of this gene polymorphism was significantly associated with increased risk of obesity by approximately four times in Thai girls, after adjusting for confounding factors. Thus, it is plausible that the genetic variant of *APLN* associated with the susceptibility of obesity may influence the apelin and its receptor system, which controls feeding behaviour. To our knowledge, no previous studies have investigated the association between the *APLN* T-1860C polymorphism and obesity in children, whereas only two previous studies in Chinese women [11] and Egyptian women [32] had investigated the relationship of the *APLN* rs3115757 variant with obesity phenotypes. Furthermore, our findings showed that the effect of the *APLN* T-1860C polymorphism was evident in females and not in males. However, the mechanisms underlying obesity may be different between male and female; the findings demonstrated that this sex-specific effect may be due to hormonal influences on gene expression and regulation or other environmental factors that are related to gender [33]. In this study, the age of participants is in the onset of puberty characterized by changes in the gonadotropin-releasing hormone (GnRH)-gonadotropin axis and previous studies in early pubertal girls suggest that excessive weight is associated with a blunted sleep-related rise of luteinizing hormone (LH) [34]. Furthermore, apelin also plays a significant role in the regulation of hypothalamic-pituitary-gonad (HPG) axis [35]. Notably, previous studies had revealed the sex-specific effect in *APLN* and other genes. In a genome-wide linkage study performed in non-Hispanic whites and African Americans, a sex-specific linkage was observed for obesity-related phenotypes [36]. Leptin receptor gene (*LEPR*) polymorphism was related to obesity in women but not in men [37]. Therefore, genetic determinants of obesity may differ between females and males. However, there is a lack of information in the literature regarding the relationship between the *APLN* T-1860C polymorphism and childhood obesity that can be compared with our findings. Although the pathogenesis of obesity is complex and not well understood, our investigation proposes that the *APLN* T-1860C polymorphism exerted a sex-specific effect on obesity in Thai children. Therefore, the *APLN* T-1860C polymorphism should be studied prospectively in a wider population to fully understand the mechanism underlying the relationship of this gene polymorphism in the etiopathology of obesity, especially in children.

The *APLNR* G212A polymorphism (rs11544374) at the 5′ untranslated region was analysed in our study and the minor allele frequency (MAF) for this SNP in Thai population (13.5%) paralleled to the previous studies which represented the MAF from Han Chinese (19.1%) [10], Vietnamese population (14.8%), East Asian (16.1%) and Danish population (15.0%) [38]. The functional role of the *APLNR* G212A polymorphism at the 5′ untranslated region has not yet been identified, and the fact that the presence of the A allele is associated with lower cardiovascular risk in an Italian population [12] consequently suggests that it may be profitable in obesity. Therefore, our study was interested in examining links of the *APLNR* G212A polymorphism with apelin concentration and obesity phenotypes among Thai children. However, the current findings among Thai children found that neither apelin concentration nor obesity phenotypes were related to the *APLNR* G212A polymorphism. However, these results are inconsistent with the findings of Kotanidou et al. [7] on Greek children and adolescents. The authors suggested that the 212A allele frequency may have a protective influence on apelin and its receptor system in childhood obesity since the presence of the 212A allele in obese youth was associated with higher circulating apelin levels. Likewise, another study on the Turkish population reported that patients with coronary artery disease seem to have lower apelin levels and higher G allele frequency [13]. In contrast, Mishra et al. studied an Indian population and demonstrated that overrepresentation of the A allele was related to reduced apelin levels in patients with high-altitude pulmonary oedema and healthy highland natives [39]. However, relatively few studies have observed a correlation between *APLNR* G212A polymorphism with circulating apelin level and obesity phenotypes. Regarding the reasons for discrepant results between the present report and previous studies, these may result from different genetic backgrounds, environmental conditions, population stratification or selection. Therefore, our findings suggest that *APLNR* G212A polymorphism may not be involved in circulating apelin concentration and obesity among Thai children. Furthermore, relationships between this polymorphism and the apelin concentration or obesity phenotypes are still not clearly understood. These relationships should be confirmed with further studies performed in larger populations and other regions of the *APLNR* gene.

Notably, there are some limitations to our study. Firstly, the distribution of body fat and its visceral deposits were not directly assessed using computed tomography. Secondly, this study did not evaluate the circulating insulin level and homeostatic model assessment for insulin resistance (HOMA-IR). Thirdly, this study did not assess the relationships between obesity and diet or family background. Finally, we did not analyse the entire *APLN* and *APLNR* genes with obesity. Further studies should replicate our findings using larger sample sizes.

## Conclusion

The present study on Thai children observed that the apelin concentrations of obese children were lower than those of normal weight children and that apelin concentrations were related to certain cardiometabolic parameters. Although no correlations between apelin concentration with *APLN* T-1860C or *APLNR* G212A polymorphisms were observed, there was an association between the *APLN* T-1860C polymorphism and obesity in female children. Therefore, the genetic polymorphism of *APLN* T-1860C and apelin concentration appeared to affect the susceptibility to obesity. As such, these findings will help to understand the pathogenesis of obesity, especially childhood. Moreover, early genotype screening may provide useful information concerning the management and assessment of obesity risk.

## Supplementary information

**Additional file 1: Table S2.** Comparison of apelin concentration between non-obese and obese children according to sex. **Table S2.** Adjusted odds ratio for *APLN* T-1860C and *APLNR* G212A polymorphisms with apelin and anthropometric-cardiometabolic variables.

## Data Availability

The data used in this study are available from the corresponding author on reasonable request.

## Abbreviations

*APLN*: Apelin gene
*APLNR*: Apelin receptor gene
BMI: Body mass index
BP: Blood pressure
CI: Confidence interval
HDL-C: High-density lipoprotein cholesterol
HWE: Hardy-Weinberg equilibrium
LDL-C: Low-density lipoprotein cholesterol
MAF: Minor allele frequency
OR: Odds ratio
PCR-RFLP: Polymerase chain reaction-restriction fragment length polymorphism
SNPs: Single-nucleotide polymorphisms
TC: Total cholesterol
TyG: Glucose index
TG: Triglycerides
