# Supplementary material for: Relationships of apelin concentration and APLN T-1860C polymorphism with obesity in Thai children
Source: BMC Pediatr. 2020 Sep 30;20:455. doi: 10.1186/s12887-020-02350-z (PMC7526109; doi:10.1186/s12887-020-02350-z)
Supplement: Supplementary file 1 — Additional file 1: Table S2. Comparison of apelin concentration between non-obese and obese children according to sex. Table S2. Adjusted odds ratio for APLN T-1860C and APLNR G212A polymorphisms with apelin and anthropometric-cardiometabolic variables. [file 12887_2020_2350_MOESM1_ESM.docx]

**Table S1** Comparison of apelin concentration between non-obese and obese children according to sex

|  | **Apelin (ng/ml)** | | | |
| --- | --- | --- | --- | --- |
| **Sex** | **Non-obese group** | **Obese group** | ***P*-value** | |
| Female  (*n* = 156) | 1.5 (0.9,2.3) | 1.1 (0.6, 1.7) | 0.038* | |
| Male  (*n* = 169) | 1.2 (0.8, 2.2) | 0.9 (0.6, 1.6) | 0.023* | |
| Data are medians with interquartile range (25^th^–75^th^ percentile) Significance level: **P* < 0.05 | | | |  |

**Table S2** Adjusted odds ratio for *APLN* T-1860C and *APLNR* G212A polymorphisms with apelin and anthropometric-cardiometabolic variables

|  | **SNP *APLN* T-1860C** | ***P*-value** | **SNP *APLNR* G212A** | ***P*-value** |
| --- | --- | --- | --- | --- |
| **Variables** | **Adjusted OR^a^ (95 % CI)** |  | **Adjusted OR^a^ (95 % CI)** |  |
| Weight (kg) | 0.99 (0.97-1.01) | 0.400 | 1.02 (0.99-1.04) | 0.174 |
| BMI (kg/m^2^) | 0.97 (0.92-1.03) | 0.339 | 1.04 (0.98-1.11) | 0.208 |
| BMI z-score | 0.89 (0.73-1.09) | 0.253 | 1.16 (0.92-1.45) | 0.202 |
| WC (cm) | 0.99 (0.96-1.01) | 0.167 | 1.02 (0.99-1.04) | 0.195 |
| Systolic BP (mmHg) | 1.01 (0.99-1.03) | 0.265 | 1.00 (0.98-1.02) | 0.959 |
| Diastolic BP (mmHg) | 1.00 (0.98-1.03) | 0.777 | 1.01 (0.98-1.03) | 0.855 |
| Apelin (ng/ml) | 1.32 (0.95-1.80) | 0.089 | 0.71 (0.49-1.06) | 0.098 |
| Glucose (mg/dl) | 0.99 (0.96-1.04) | 0.938 | 1.02 (0.98-1.06) | 0.409 |
| TyG index | 1.13 (0.66-1.93) | 0.660 | 0.99 (0.55-1.85) | 0.993 |
| TC (mg/dl) | 1.00 (0.99-1.01) | 0.947 | 1.00 (0.99-1.01) | 0.343 |
| TG (mg/dl) | 1.00 (0.99-1.01) | 0.620 | 1.00 (0.99-1.01) | 0.741 |
| HDL-C (mg/dl) | 0.99 (0.97-1.01) | 0.404 | 1.01 (0.99-1.04) | 0.297 |
| LDL-C (mg/dl) | 1.00 (0.99-1.01) | 0.858 | 1.01 (0.99-1.02) | 0.356 |
| LDL-C/HDL-C | 1.04 (0.73-1.47) | 0.844 | 1.14 (0.78-1.67) | 0.503 |
| TC/HDL-C | 1.03 (0.78-1.38) | 0.817 | 1.07 (0.77-1.47) | 0.699 |
| TG/HDL-C | 1.01 (0.83-1.22) | 0.967 | 0.96 (0.77-1.21) | 0.743 |

**^a^** Values are the odds ratio adjusted for sex, age and acanthosis nigricans

95% CI is the 95% confidence interval of the odds ratio

.
